# The Role of Extracorporeal Membrane Oxygenation and Atrial Pacing in Congenital Junctional Ectopic Tachycardia: A Case Report

**DOI:** 10.1016/j.cjcpc.2025.04.005

**Published:** 2025-04-23

**Authors:** Nicolas Mourad, Diego Marquez, Cheryl Peters, Steven Rathgeber, Sakethram Saravu Vijayashankar, Ryaan EL-Andari, Muhieldin Ahamad Muhieldin

**Affiliations:** a Department of Cardiac Surgery, BC Children’s Hospital, Vancouver, British Columbia, Canada; b Department of Cardiology and Interventional Cardiology, BC Children’s Hospital, Vancouver, British Columbia, Canada; c Division of Cardiac Surgery, University of Alberta, Edmonton, Alberta, Canada

**Congenital junctional ectopic tachycardia is a rare arrhythmia, typically refractory to medical therapy and associated with high mortality and morbidity rates. Data from large electrophysiology centers have reported less than 100 cases over the preceding 4 decades, with a 35% mortality rate.****^1^** **Up to 16% of patients initially present with congestive heart failure.****^1^** **Although most of these cases are managed medically, some patients develop hemodynamic instability, leading to more urgent and invasive procedures. We present a case of an infant with congenital junctional ectopic tachycardia requiring medical management, venoarterial extracorporeal membrane oxygenation, and a single-chamber permanent pacemaker to manage arrhythmia-induced cardiogenic shock.**

## Case Report

A previously healthy 7-week-old term male infant with normal fetal dating ultrasound and echocardiography at 20 weeks of gestation was found limp and cyanotic by his mother. She immediately started cardiopulmonary resuscitation (CPR), and an ambulance arrived 10 minutes later, with paramedics continuing CPR for a total of 20 minutes, followed by return of spontaneous circulation, as they transported him to the nearest emergency department.

On examination at a small peripheral emergency department, the patient weighed 5.03 kg, was poorly perfused with delayed capillary refill, appeared pale and mottled, was in respiratory distress, and had altered consciousness with a heart rate of 240 beats per minute (bpm) and a borderline narrow complex QRS of 80 ms. Two doses of adenosine, 0.5 mg (0.1 mg/kg) and 1 mg (0.2 mg/kg), and one dose of amiodarone, 25 mg (5 mg/kg), were administered with no response. Three direct current cardioversions also failed to terminate the tachyarrhythmia. He was intubated and mechanically ventilated, after which he was transferred to our tertiary center (BC Children’s Hospital, British Columbia, Vancouver, Canada). The workup of this patient is described in Supplemental Appendix S1. Notably, an echocardiogram showed an ejection fraction (EF) of 20%, a dilated left ventricle and atrium, and moderate mitral regurgitation.

**Figure 1:**
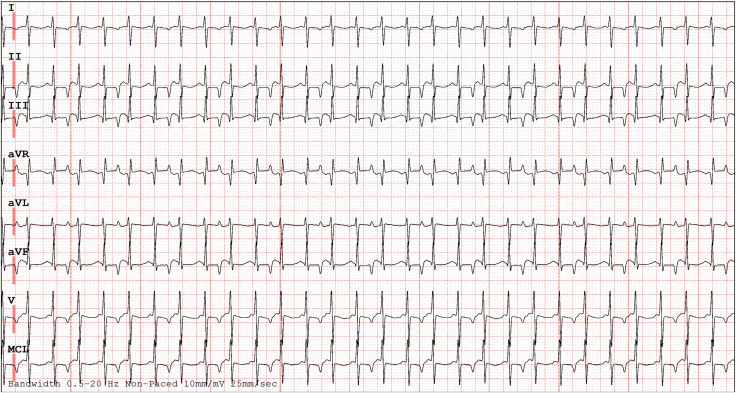
Electrocardiogram showing narrow complex (QRS: 80 ms) tachycardia with 2:1 ventricular-atrial (VA) conduction. The *P* waves are suggestive of a low to high atrial activation secondary to intermittent VA conduction.

After amiodarone loading with 5 mg/kg intravenously over 60 minutes, there was minimal arrhythmia response with significant hemodynamic compromise, so venoarterial extracorporeal membrane oxygenation (ECMO) was initiated 14 hours after hospitalization. Figure 1 illustrates the electrocardiogram obtained after ECMO initiation. Amiodarone loading continued for a maintenance infusion rate of 7.5 mcg/kg/min, and 1 mcg/kg/h of dexmedetomidine was maximized for sedation, reducing the junctional rate to 120 bpm. The patient underwent left atrial decompression for left atrial hypertension via transeptal puncture and balloon septostomy 24 hours after ECMO initiation. Recovery over the next 4 days was slow with heart rate ranging between 140 and 160 bpm, which was mostly junctional. Thus, ivabradine 0.5 mg (0.1 mg/kg) twice daily was added orally, which decreased the junctional rate to a range of 90-105 bpm. Slow ventricular recovery was noted. Unfortunately, the ECMO run was complicated by a right posterior cerebral artery infarct, necessitating decannulation on day 4 (total ECMO duration: 97 hours) before adequate ventricular function recovery. Significant cardiac output improvement was noted when atrioventricular (AV) synchrony was established with esophageal atrial pacing over the junctional rate. Because of a bleeding diathesis, temporary epicardial wires could not be placed, and the patient was decannulated with esophageal atrial pacing, which was continued for 18 hours till the bleeding diathesis was controlled. Atrial pacing for a period of 1 hour, followed by 2- to 3-minute breaks to rest esophageal tissue, was performed to maintain AV synchrony. Because of the persistence of junctional rhythm and continued requirement for AV synchrony, a permanent epicardial atrial pacemaker was then placed. During the initial 2 weeks after decannulation, the patient was mostly in an atrially paced rhythm. Sinus rhythm was slowly established over the junctional rate. Care was deintensified, and the patient was transferred to the ward. He spent 27 days in the hospital for stroke rehabilitation and sedation weaning. At discharge, he was predominantly in sinus rhythm, his heart rate ranged between 108 and 132 bpm, and the echocardiogram showed recovered ventricular function, an EF of 64%, and resolved mitral regurgitation.

At 6-week follow-up, he remained on amiodarone 4.4 mg/kg per oral once daily and ivabradine 150 mcg/kg per oral 3 times daily. He was growing and feeding well. His pacemaker, set to 70 bpm, showed pacing 1.4% of the time and an underlying junctional rhythm with intermittent sinus beats at 110 bpm. His echocardiogram revealed normal biventricular systolic function and an EF of 65%. Finally, his comprehensive cardiac gene panel was negative, and he did not have familial junctional ectopic tachycardia or any siblings who required further testing.

## Discussion

Coumel^2^ in 1976 first described the first series of 7 infants with supraventricular tachycardia and AV dissociation. Presently, junctional tachycardia in children is seen as 2 distinct entities: the common postoperative junctional ectopic tachycardia and the rare congenital junctional ectopic tachycardia (CJET). Increased automaticity of the AV nodal tissue or the His-Purkinje system is the proposed mechanism of the arrhythmia. This is supported by the fact that there is often ventricular-atrial conduction in CJET, with a narrow QRS complex, and nontermination with adenosine or cardioversion.^3^ Familial incidences of CJET have been demonstrated over the years with multiple gene deletions like the angiotensin-converting enzyme insertion/deletion and troponin I–interacting kinase being implicated in predisposition to CJET.^3^

Medical management is the mainstay of initial treatment for most patients, with amiodarone being the first-line agent, often used in combination with ivabradine, procainamide, flecainide, esmolol, or propranolol.^3^ Of note, intravenous amiodarone has been associated with a risk of hypotension, bradycardia, and ventricular tachycardia.^3^ Critical therapies in addition to antiarrhythmic medications aim to reduce catecholamines. Commonly used methods are electrolyte repletion, temperature control, sedation, treating dehydration and pain, and treating acidosis.^3^

Given the scarce literature on CJET cases, there are no set management algorithms, requiring quick clinical decisions as patients often rapidly decompensate and do not easily respond to treatment. In our case, the patient arrested at home, had CPR for 20 minutes, and subsequently required ECMO initiation and atrial pacing to maintain AV synchrony. This patient was paced through the esophagus for a prolonged period before an epicardial system was placed. This epicardial system is now used minimally, and in retrospect, we might have achieved satisfactory results by placing a temporary epicardial pacemaker.

Two previous case reports described the use of ECMO in hemodynamically unstable patients with CJET. Darst and Kaufman^4^ reported a 2-month-old patient in cardiogenic shock secondary to CJET requiring 3 days of ECMO support, whereas Mudery et al.^5^ reported a 3-month-old patient with heart failure and cardiogenic shock secondary to CJET requiring 6 days of ECMO support.

Our patient was placed on ECMO support for a total of 4 days, after which he required an atrial pacing to achieve AV synchrony while on antiarrhythmic medications. He then did well after discharge, illustrating the vital role and benefit of ECMO, and pacing, for patients with CJET who are hemodynamically unstable.

Mariani et al.^6^ have previously described the role of ECMO in acting as a safe rescue therapy for hemodynamic instability and in supporting aggressive medical treatments in pediatric tachyarrhythmias. Thus, when a patient presents with CJET and hemodynamic instability, considerations should be made regarding transferring to a center with ECMO support.

Finally, discussions must also revolve around the possibility of atrial pacing to provide AV synchrony and optimize cardiac output. This proved to be of benefit to our patient whose biventricular systolic function normalized by discharge. The atrial pacing was vital in the initial recovery phase but was needed less once good rate control and return of ventricular function was achieved.

## Conclusions

We present a case of CJET where ECMO was used during hemodynamic collapse, and atrial pacing was used to maintain AV synchrony. While uncommon, tachycardia-induced heart failure leading to hemodynamic instability and cardiogenic shock remains a risk in these patients, thereby requiring prompt decision-making in using ECMO support to avoid further deterioration.

**Novel Teaching Points**

• ECMO may be required in cases of CJET with hemodynamic instability.
• Atrial pacing may be required in the initial phase of recovery from tachycardia-induced cardiomyopathy in newborns to maintain AV synchrony, and while not a regular practice, may be of benefit in cases of refractory CJET.
