# Supplementary material for: The Role of Extracorporeal Membrane Oxygenation and Atrial Pacing in Congenital Junctional Ectopic Tachycardia: A Case Report
Source: CJC Pediatr Congenit Heart Dis. 2025 Apr 23;4(5):274–6. doi: 10.1016/j.cjcpc.2025.04.005 (PMC12835902; doi:10.1016/j.cjcpc.2025.04.005)
Supplement: Supplemental Material [file mmc1.docx]

**The Role of Extracorporeal Membrane Oxygenation and Atrial Pacing in Congenital Junctional Ectopic Tachycardia: A Case Report**

**Supplemental Material**

**Supplemental Materials**

Supplemental Appendix S1: Work-up

In the pediatric intensive care unit, an echocardiogram identified normal structural anatomy but severely reduced biventricular systolic function with an ejection fraction (EF) of 20%, a dilated left ventricle and atrium, and moderate mitral regurgitation, indicating a long-standing cardiomyopathy likely secondary to tachycardia. An atrial wire study using an esophageal pacing catheter revealed incessant tachycardia at 200-240 bpm with subtle cycle length variations. Adenosine administration showed continued tachycardia with AV dissociation, ruling out atrial tachycardia, AV reciprocating tachycardia, and AV nodal re-entry tachycardia. The differential diagnosis included fascicular ventricular tachycardia or CJET. Verapamil 150mcg/kg IV was administered over 30 minutes but did not significantly alter the tachycardia. Atrial overdrive pacing accelerated the tachycardia, with similar QRS morphology and axis, confirming CJET. Given its high response rate in the literature, amiodarone loading was thus initiated.
